# Chromosome-level genome assembly of the humpback puffer, *Tetraodon palembangensis*

**DOI:** 10.46471/gigabyte.17

**Published:** 2021-04-01

**Authors:** Rui Zhang, Chang Li, Mengjun Yu, Xiaoyun Huang, Mengqi Zhang, Shanshan Liu, Shanshan Pan, Weizhen Xue, Congyan Wang, Chunyan Mao, He Zhang, Guangyi Fan

**Affiliations:** ^1^ BGI-Qingdao, BGI-Shenzhen, Qingdao 266555, China; ^2^ Department of Biology, Hong Kong Baptist University, Hong Kong, China

## Abstract

The humpback puffer, *Tetraodon palembangensis*, is a poisonous freshwater pufferfish species mainly distributed in Southeast Asia (Thailand, Laos, Malaysia and Indonesia). The humpback puffer has many interesting biological features, such as inactivity, tetrodotoxin production and body expansion. Here, we report the first chromosome-level genome assembly of the humpback puffer. The genome size is 362 Mb, with a contig N50 value of ∼1.78 Mb and a scaffold N50 value of ∼15.8 Mb. Based on this genome assembly, ∼61.5 Mb (18.11%) repeat sequences were identified, 19,925 genes were annotated, and the function of 90.01% of these genes could be predicted. Finally, a phylogenetic tree of ten teleost fish species was constructed. This analysis suggests that the humpback puffer and *T. nigroviridis* share a common ancestor 18.1 million years ago (MYA), and diverged from *T. rubripes* 45.8 MYA. The humpback puffer genome will be a valuable genomic resource to illustrate possible mechanisms of tetrodotoxin synthesis and tolerance.

## Data description

### Background and context

**Figure 1.**
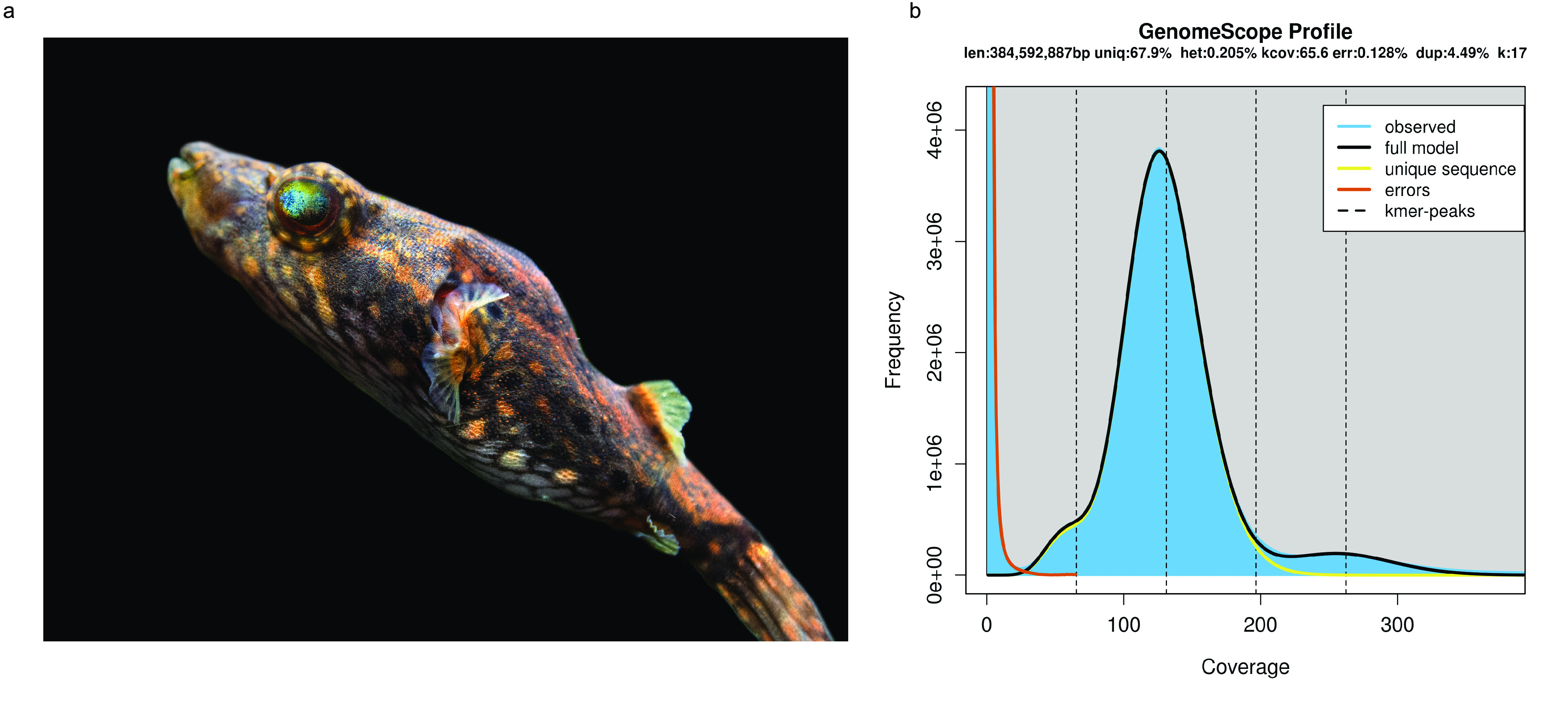
*Tetraodon palembangensis.* (a) Photograph of *Tetraodon palembangensis.* Photo courtesy of Preston Swipe, Aquatic Arts LLC. (b) The 17-mer depth distribution of the stLFR reads. The estimated genome size is 385 Mb; heterozygosis is 0.21%.

The humpback puffer, *Tetraodon palembangensis* (NCBI Taxonomy ID: 1820603, Fishbase ID: 25179), also known as *Pao palembangensis*, is widely distributed in Southeast Asia and prefers to live in alkalescent, warm (24–28°), and slow-flowing rivers (Figure 1a) [1]. The female and male humpback puffers have a similar body size, but the male’s rear hump is much bigger than that of the female [2]. The humpback puffer is a popular ornamental fish because of its beautiful skin colouration and patterns. Unlike other species of predatory pufferfish, the humpback puffer is lazy and will not initiatively look for food [1]. Furthermore, its body contains a deadly toxin, known as tetrodotoxin (TTX), and it can swell up to three times its normal size as a defense mechanism when threatened [1]. Previous studies have shown that the toxicity of the humpback puffer varies greatly between seasons [3]. The wild population of humpback puffer has declined in recent years owing to the destruction of suitable habitat caused by climate change and overfishing [4].

With these biological characteristics and a small genome size, the humpback puffer is an ideal species for genetic study [5]. It is also a species in the Fish10K Genome Project, a subproject of the Earth BioGenome Project, which aims to sample, sequence, assemble and analyse the genomes of 10,000 fish species [6]. In this study, we provide a chromosome-scale genome assembly of the humpback puffer. This assembly will be valuable for further study of mechanisms, such as tetrodotoxin synthesis and expansion defense. Comparative genomic analysis will help us to better understand the phylogenetic evolution and special gene families of the Tetraodontidae.

## Methods

**Figure 2.**
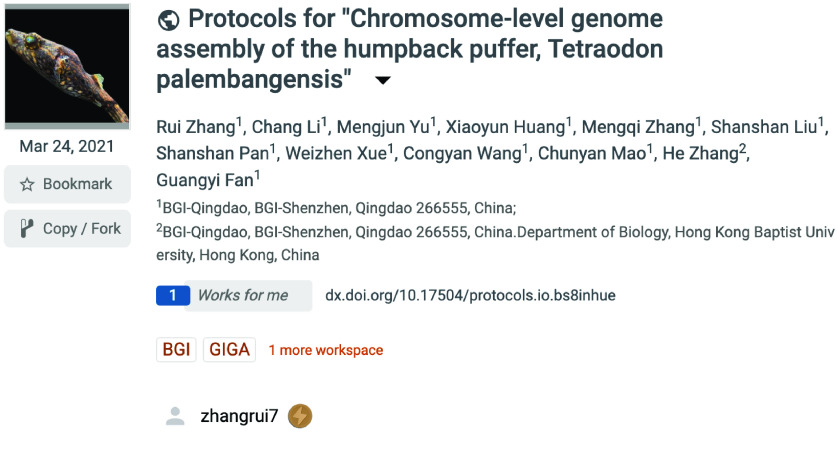
Protocols.io collection for the chromosome-level genome assembly of the humpback puffer, *Tetraodon palembangensis*. https://www.protocols.io/widgets/doi?uri=dx.doi.org/10.17504/protocols.io.bs8inhue

All methods used to isolate DNA/RNA, construct libraries, and conduct genomic sequencing are available in a protocols.io collection (Figure 2 [7]).

### Sample collection and sequencing

The sample (CNGB ID: CNS0224034) used in this study was an adult humpback puffer bought from the YueHe Flower-Bird-Fish market in Guangzhou Province, China. DNA and RNA were both isolated from blood following published protocols [8, 9]. Then, a paired-end single tube long fragment reads (stLFR) library and an RNA library were constructed according to the protocol published by Wang *et al.* [10]. A Hi-C library was constructed from blood according to the protocol published by Huang *et al.* [11]. These three libraries were then sequenced on the BGISEQ-500 platform (RRID:SCR_017979) [12]. A Nanopore library was constructed with DNA isolated from blood using the QIAamp DNA Mini Kit (Qiagen) [13] and sequenced on the GridION platform (RRID:SCR_017986) [14]. In total, we obtained 146 Gb (∼312×) raw stLFR data, 21 Gb raw RNA data, 19 GB (∼49×) raw Hi-C data, and 12 GB (∼32×) raw Nanopore data (Table 1).

Raw stLFR reads were subjected to quality control to improve the assembly quality. Firstly, we obtained co-barcoding information from the last 42 bases of read 1 and deleted the last 42 bases. Then SOAPnuke (v1.6.5, RRID:SCR_015025) [15] was used to filter remaining reads, using the parameters: “–M 1 –d –A 0.4 –n 0.05 –l 10 –q 0.4 –Q 2 –G –5 0” [16]. Finally, 62 Gb (152×) clean data were retained for further assembly. Raw RNA reads were also filtered by SOAPnuke using the parameters: “–M 1 –A 0.4 –n 0.05 –l 10 –q 0.4 –Q 2 –G –5 0”, generating 20 Gb clean data. Raw Hi-C data were produced with a quality control using HiC-Pro (v. 2.8.0) [16] with default parameters. This generated 5.4 Gb validated data, which accounted for 28.81% of all data (Table 1).

**Table 1:** Statistics of sequencing data.

Libraries	Read lengths (bp)	Raw data		Valid data	
		Total bases (Gb)	Sequencing depth (×)	Total bases (Gb)	Sequencing depth (×)
stFLR	PE100	120.4	311.69	62.6	162.60
RNA	PE 100	20.6	–	20.0	–
Hi-C	PE 100	19.01	49.43	5.4	14.04
Nanopore	CN50: 32 kb	12.3	31.98	–	–

Sequencing depth = total bases/genome size, where the genome size is the result of *k*-mer estimation in Table 2. Abbreviations: CN5, contig N50; PE, paired end.

### Genome assembly

**Table 2:** Statistics of 17-mer analysis.

*k*-mer	*k*-mer number	*k*-mer depth (×)	Heterozygosity (%)	Genome size (bp)
17	48,458,703,762	126	0.205	384,592,887

Jellyfish (v2.2.6, RRID:SCR_005491) was used to count *k*-17mers of all clean stLFR reads [17]. Genomescope [18] was used to estimate the humpback puffer genome size at about 385 Mb (Table 2 and Figure 1b).

The genome size, *G*, was defined as *G* = *K*_num_∕*K*_depth_, where the *K*_num_ is the total number of *k*-mers, and *K*_depth_ is the most frequently occurring frequency.

To assemble the humpback puffer genome, we firstly converted the format of clean stLFR reads, then used Supernova (v. 2.0.1, RRID:SCR_016756) to perform the draft assembly. Then, we used GapCloser (v. 1.12, RRID:SCR_015026) [19] to fill gaps with stLFR reads. To futher improve the assembly quality, TGSgapFiller [20] was then used to re-fill gaps with Nanopore reads, and Pilon (v. 1.22, RRID:SCR_014731) [21] was used to polish the assembly twice. At this stage, the genome assembly was about 362 Mb, with 7.1-Mb scaffold N50 and 1.8-Mb contig N50 values (Table 3).

**Figure 3.**
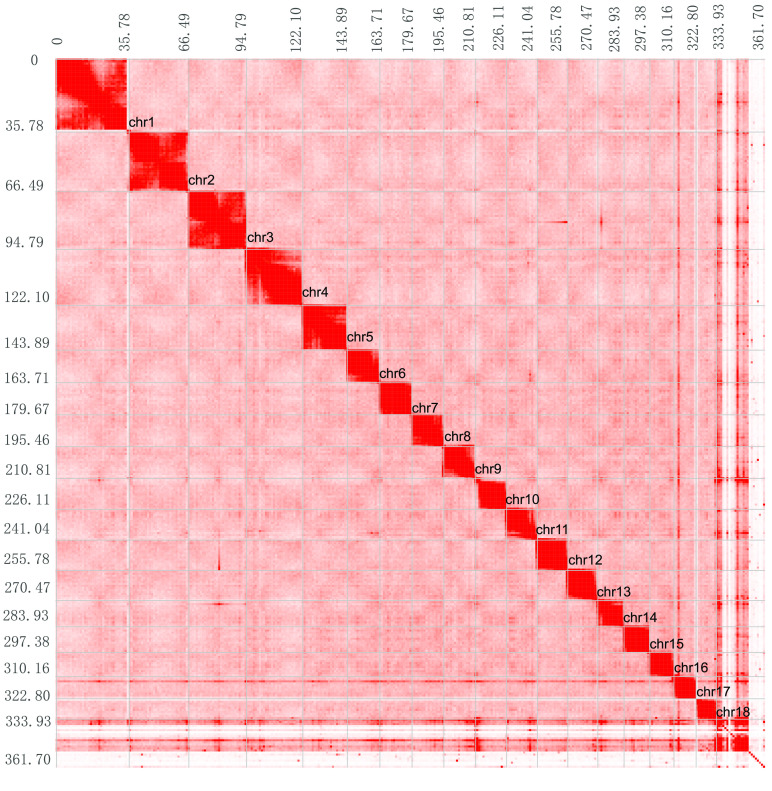
Heat map of chromosomal interaction from Hi-C reads. Grey lines show the border between chromosomes.

With the genome and validated Hi-C data from HiC-Pro, the contact matrix was generated by Juicer (v3, RRID:SCR_017226). Finally, we perfomed chromosomal-level scaffolding using the 3D de novo assembly (3D-DNA) pipeline (v. 170123) [22]. This anchored 91.2% of all sequences to 18 chromosomes, with a length ranging from 11 Mb to 35 Mb (Table 4 and Figure 3).

The karyotype differs among genuses in Tetraodontidae [23–27]. For example, *Takifugu rubripes*, *Takifugu obscurus* and *Takifugu flavidus* have 2*n* = 44 chromosomes, while *Tetraodon nigroviridis and Tetraodon fluviatilis* have 2*n* = 42 chromosomes. In addition, *Thamnaconus septentrionalis* (Monacanthidae) has 2*n* = 40 chromosomes. Thus, we defined the chromosome number of the humpback puffer to be 18, according to the apparent and logical interactions by Hi-C reads.

**Table 3:** Statistics of the draft assembly of the humpback puffer genome.

Statistics	Scaffold	Contig
Total number (#)	5291	6190
Total length (bp)	361,704,206	360,427,744
Gap (N) (bp)	1,276,462	0
Average length (bp)	68,362	58,227
N50 length (bp)	7,059,990	1,830,664
N90 length (bp)	453,057	157,209
Maximum length (bp)	19,534,197	9,842,180
Minimum length (bp)	682	48
GC content (%)	44.66	44.66

**Table 4:** Statistics of the Hi-C scaffolding of the humpback puffer genome.

Statistics	Scaffold	Contig
Total number (#)	5366	6435
Total length (bp)	361,698,760	360,427,744
Gap (N) (bp)	1,271,016	0
Average length (bp)	67,406	56,011
N50 length (bp)	15,808,960	1,794,775
N90 length (bp)	11,014,520	117,115
Maximum length (bp)	34,916,285	9,792,502
Minimum length (bp)	682	48
GC content (%)	44.66	44.66

### Genomic annotation

**Figure 4.**
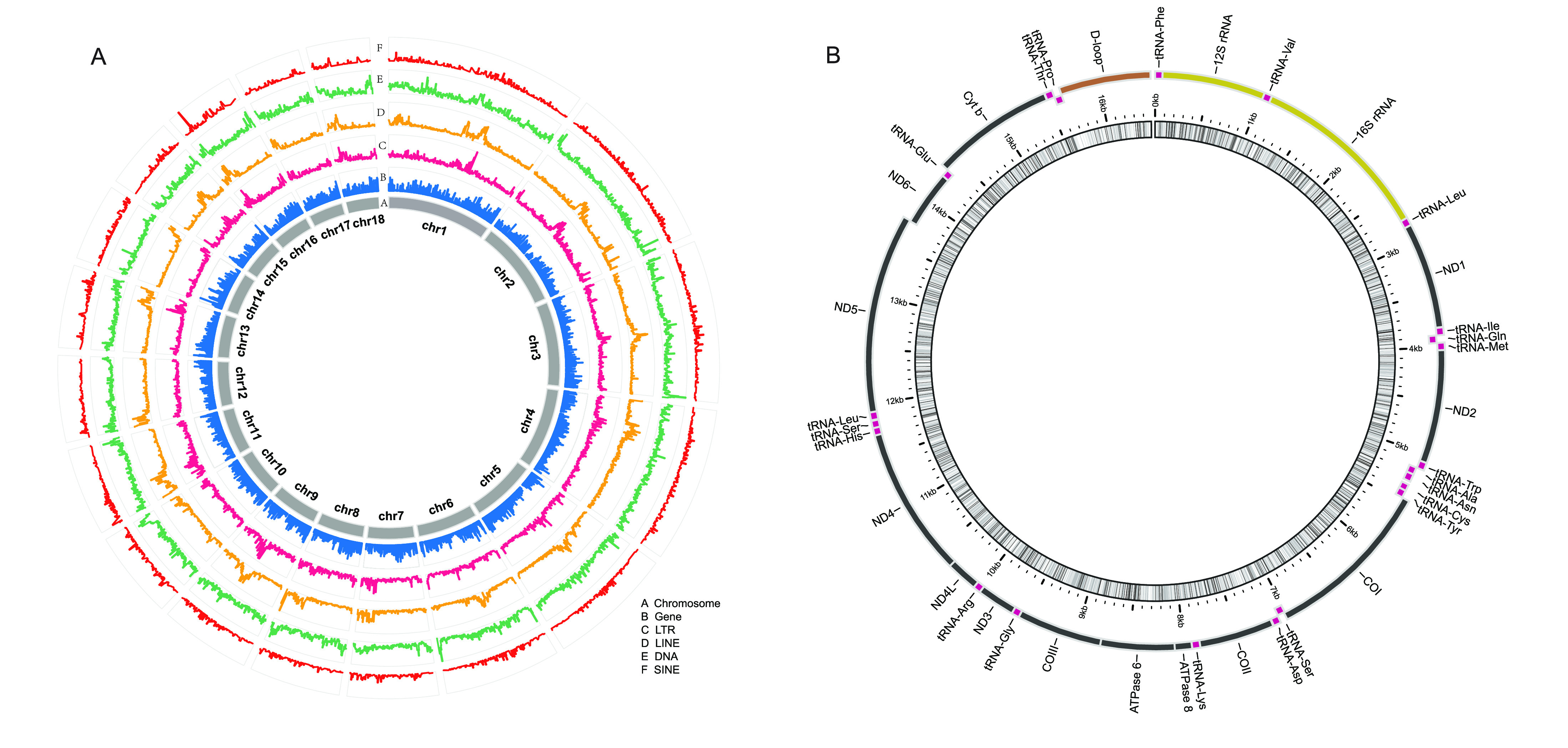
Annotation of the *Tetraodon palembangensis* genome. (a) Basic genomic elements of the genome. LTR, long terminal repeat; LINE, long interspersed nuclear elements; SINE, short interspersed elements. (b) Physical map of mitochondrial assembly.

Two methods were used to annotate repetitive sequences. Firstly, we aligned the genome to the Repbase library by TRF (v.4.09) [28]. RepeatMasker (v. 3.3.0, RRID:SCR_012954) and RepeatProteinMask (v. 3.3.0) [29] were then used to predict and classify the repetitive sequences. Secondly, we constructed a repeat library using RepeatModeler (v1.0.8, RRID:SCR_015027) and classified the transposable elements (TEs) with RepeatMasker (v. 3.3.0) [29]. The results of both methods were amalgamated to give a total of 65 Mb repeat sequences and 59 Mb TEs, accounting for 18.11% (Table 5 and Figure 4a) and 16.62% of the entire genome, respectively (Table 6 and Figure 4a).

Using the clean, reformatted stLFR reads, the mitochondrial genome of the humpback puffer was assembled using MitoZ [30] with default parameters. Mitochondrial genes were annotated using the MitoAnnotator tool of the mitofish pipeline [31] (Figure 4b). For gene structural annotation, we performed *de novo* prediction using AUGUSTUS (v3.1, RRID:SCR_008417) [32], GlimmerHMM (v3.0.4, RRID:SCR_002654) [33], and Genscan (RRID:SCR_013362) [34]. We also used TRINITY (v2.8.5, RRID:SCR_013048) [35] to assemble a draft transcriptome with clean RNA reads, then HISAT2 (v2.1.0, RRID:SCR_015530)-StringTie (v1.3.4, RRID:SCR_016323) [36] and PASA (v2.3.3, RRID:SCR_014656)-TransDecoder (RRID:SCR_017647) [37] to predict transcripts. GeneWise (v2.4.1, RRID:SCR_015054) [38] was used for homologous annotation, with protein data obtained from the National Center for Biotechnology Information (NCBI) database for the following eight species: *Danio rerio* (NCBI, GenBank ID:50), *Cynoglossus semilaevis* (NCBI, GenBank ID:11788), *Gasterosteus aculeatus* (NCBI, GenBank ID:146), *Gadus morhua* (NCBI, GenBank ID:2661), *Larimichthys crocea* (NCBI, GenBank ID:12197), *Oreochromis niloticus* (NCBI, GenBank ID:197), *Oryzias latipes* (NCBI, GenBank ID:542), and *Takifugu rubripes* (NCBI, GenBank ID:63). Finally, these three types of evidence were integrated using EVidenceModeler (v1.1.1, RRID:SCR_014659) [39], generating 19,925 nonredundant coding genes, each containing an average of 11 exons and a 1945 bp coding region (Table 7).

For gene function annotation, we aligned the 19,925 genes to the TrEMBL (UniProtKB, RRID:SCR_004426) [40], Swissprot [41], Kyoto Enyclopedia of Genes and Genomes (KEGG, RRID:SCR_012773) [42], Gene Ontology (GO, RRID:SCR_002811) [43] and InterProScan (RRID:SCR_005829) [44] databases. Overall, 90.1% of all genes were able to be functionally annotated (Table 8 and Figure 3).

**Table 5:** Statistics of repeat sequences.

Type	Repeat size (bp)	% of genome
TRF	9,050,571	2.52
RepeatMasker	34,142,529	9.50
RepeatProteinMask	17,674,660	4.92
De novo	57,492,865	16.00
Total	65,080,476	18.11

**Table 6:** Statistics of transposable elements (TEs).

Type	RepBase TEs		TE proteins		*De novo*		Combined TEs	
	Length (bp)	% of genome	Length (bp)	% of genome	Length (bp)	% of genome	Length (bp)	% of genome
DNA	12,412,491	3.45	1,086,262	0.30	16,089,219	4.48	22,470,373	6.25
LINE	18,430,929	5.13	13,695,154	3.81	29,418,621	8.19	33,421,782	9.30
SINE	524,061	0.15	0	0.00	289,252	0.08	789,086	0.22
LTR	5,393,600	1.50	2,906,451	0.81	12,758,934	3.55	15,803,098	4.40
Other	8,290	0.00	228	0.00	0	0.00	8,518	0.00
Unknown	0	0.00	0	0.00	3,202,764	0.89	3,202,764	0.89
Total	34,142,529	9.50	17,674,660	4.92	55,052,617	15.32	59,729,335	16.62

Abbreviations: LINE, long interspersed nuclear elements; LTR, long terminal repeats; SINE, short interspersed nuclear elements; TE, transposable elements.

**Table 7:** Statistics of the predicted genes in the humpback puffer genome.

	Gene set	Gene number	Average transcript length (bp)	Average CDS length (bp)	Average intron length (bp)	Average exon length (bp)	Average exons per gene
Homolog	*Cynoglossus semilaevis*	19,686	9136.12	1715.14	856.1	177.4	9.67
		*Danio rerio*	19,348	15,066.80	1577.39	1718.92	178.28	8.85
		*Gadus morhua*	20,361	7040.85	1441.77	744.62	169.23	8.52
		*Gasterosteus aculeatus*	26,630	6896.85	1474.53	686.88	165.79	8.89
		*Larimichthys crocea*	21,220	9425.27	1690.06	902.2	176.53	9.57
		*Oreochromis niloticus*	24,562	9494.62	1789.15	829.18	173.82	10.29
		*Oryzias latipes*	23,332	8859.46	1467.62	962.5	169.08	8.68
		*Takifugu rubripes*	19,635	7762.47	1645.04	707.22	170.47	9.65
*De novo*	Augustus	21,662	7149.08	1725.00	659.42	186.98	9.23
		Genscan	25,933	9855.53	1791.43	990.72	196.01	9.14
		GlimmerHMM	99,722	1192.96	594.53	378.42	230.31	2.58
Transcript	Pasa & Transdecoder	33,965	4856.71	1186.88	558.33	156.73	7.57
		Hisat & Stringtie	31,664	5551.59	1303.52	608.39	163.3	7.98
EVM		19,925	9418.80	1945.48	757.65	179.08	10.86

The EVM gene set contains the integrated result of *De novo* gene predictions, homolog gene predictions and transcript annotation by EVM software.

**Table 8:** Statistics of the functional annotation.

Database	Number of genes	Gene functionally annotated (%)
Total	20,057	100.00
SwissProt	17,333	86.42
KEGG	16,182	80.68
TrEMBL	18,037	89.93
Interpro	17,108	85.30
Overall	18,064	90.06

### Genome evolution

**Figure 5.**
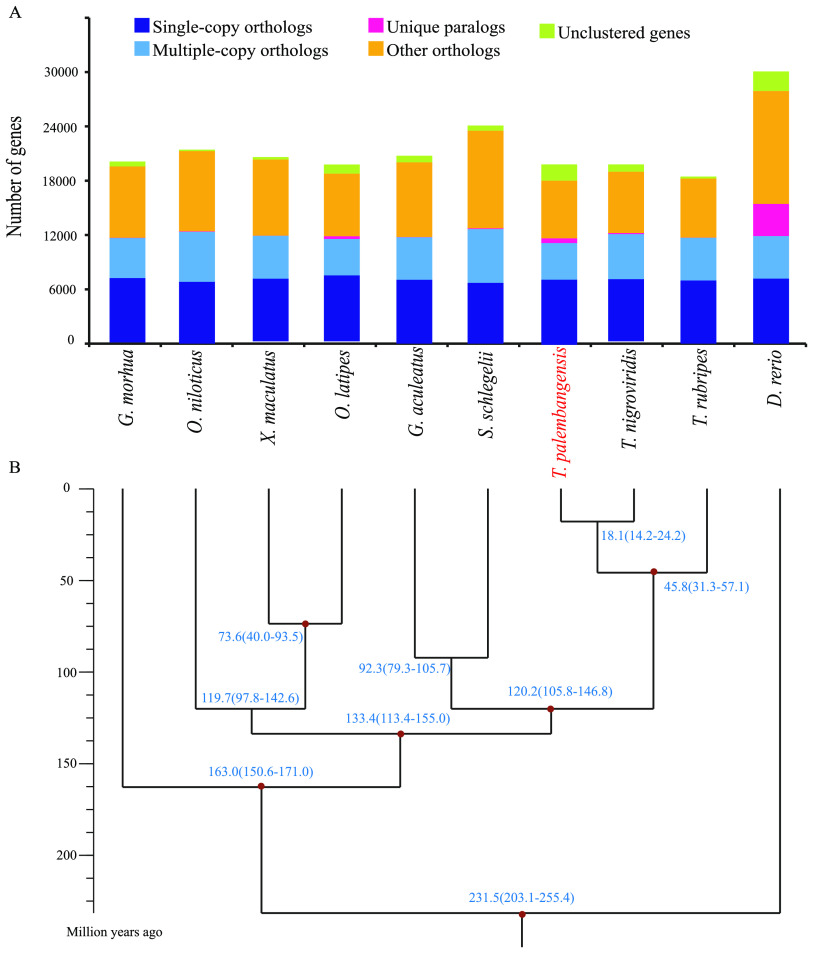
Comparative analysis of the *Tetraodon palembangensis* and nine teleosts. (a) Clustering of gene families. (b) Phylogenetic tree constructed with the single-copy gene families. The fossil correction nodes in the tree are highlighted by red dots.

To study the evolutionary status of humpback puffer among bony fish species, we clustered gene families by alignment using protein sequences of the humpback puffer and nine other teleosts (*Xiphophorus maculatus*, *Gasterosteus aculeatus*, *Sebastes schlegelii*, *Oryzias latipes*, *Gadus morhua*, *Oreochromis niloticus*, *Tetraodon nigroviridis*, *Danio rerio*, and *Takifugu rubripes*) using the TreeFam v0.50 pipeline [45]. Protein-coding genes sequences for all of these species were downloaded from NCBI, except for *S. schlegelii*  [46], which was obtained from the China National Genebank Nucleotide Sequence Archive (CNSA; Accession ID: CNP0000222). To improve analysis quality, we removed genes either with frameshifts, or less than 50 amino acids, as well as redundant copies, only keeping the longest transcripts for comparative genomic analysis. A total of 21,022 gene families were identified, of which 40 gene families were unique to the humpback puffer (Table 9 and Figure 5a).

Of all 21,022 gene families, we identified 4461 single-copy protein-coding genes shared by all species. We used MUSCLE v3.8.31 [47] to align these orthologs, with default parameters. Then, the alignments were concatenated into a 3,584,782 amino acid “super alignment matrix”. Based on this matrix, a phylogenetic tree was constructed using RAxML v8.2.4 [48], with the best amino acid substitution model-JTT. Clade support was assessed using a bootstrapping algorithm with 1000 alignment replicates (Figure 5b). Next, we calculated the divergence time among these teleosts using the MCMCTree tool included in PAML (v4.7a, RRID:SCR_014932) [49], with parameters of “–rootage 500 -clock 3 -alpha 0.431879”. The fossil correction time (Table 10) was obtained from Timetree [50]. The result showed that the humpback puffer and *T. nigroviridis*, two species belonging to the same genus, shared a common ancestor 18.1 millon years ago (MYA) and diverged from *T. rubripes* 18.1 MYA (Figure 5b).

**Table 9:** Statistics of gene family clustering.

Species	Total number of genes	Number of unclustered genes	Number of gene families	Number of unique families	Average number of genes per family
*D. rerio*	30,067	2171	18,635	735	1.5
*G. aculeatus*	20,756	728	15,995	11	1.25
*G. morhua*	19,987	525	15,650	11	1.24
*S. schlegelii*	24,094	558	16,991	30	1.39
*O. latipes*	19,535	984	14,873	71	1.25
*O. niloticus*	21,431	160	15,811	13	1.35
*T. nigroviridis*	19,544	805	14,916	50	1.26
*T. palembangensis*	19,796	690	15,830	40	1.21
*T. rubripes*	18,459	207	14,733	6	1.24
*X. maculatus*	20,356	271	16,446	3	1.22

**Table 10:** Fossil correction time used in divergence analysis.

Taxon 1	Taxon 2	Fossil time (MYA)	Minimum (MYA)	Maximum (MYA)
*Takifugu rubripes*	*Tetraodon nigroviridis*	52	42	59
*Takifugu rubripes*	*Gadus morhua*	148	141	170
*Oryzias latipes*	*Xiphophorus maculatus*	93	76	111
*Oryzias latipes*	*Gasterosteus aculeatus*	128	105	154
*Oryzias latipes*	*Danio rerio*	229.9	204.5	255.3

## Data validation and quality control

To demonstrate the quality of genome assembly and gene set, we performed a qulity evaluation using the actinopterygii_odb10 database from Benchmarking Universal Single-Copy Orthologs (BUSCO v.4.1.2, RRID:SCR_015008) [51]. The results showed that 95.7% and 90.7% complete BUSCOs were covered by the genome assembly and gene set, respectively (Table 11).

**Table 11:** Statistics of the BUSCO assessment.

Types of BUSCOs	Genome assembly		Gene set	
	Number	Percentage (%)	Number	Percentage (%)
Complete BUSCOs	3486	95.7	3303	90.7
Complete and single-copy BUSCOs	3427	94.1	3252	89.3
Complete and duplicated BUSCOs	59	1.6	51	1.4
Fragmented BUSCOs	45	1.2	96	2.6
Missing BUSCOs	109	3.1	241	6.7
Total BUSCOs groups searched	3640	100	3640	100

## Reuse potential

We assembled the first annotated chromosome-level genome of the humpback puffer. These resources will be helpful to study the mechanism of body expansion displayed by this fish species, the synthesis mechanism and treatment of tetrodotoxin, as well as the evolution of freshwater puffer. Futhermore, the humpback puffer genome will fill a gap missing from the Fish 10K program and in the phylogenetic tree of life.

## Data Availability

We have deposited the project at CNGB Nucleotide Sequence Archive (CNSA) where the accession ID is CNP0001025. The genomic data can be obtained in *GigaScience* Database [52]. The sequencing data have been deposited at National Center for Biotechnology Information (NCBI) where the bioproject accession ID is PRJNA597275.

## List of abbreviations

bp: base pair; BUSCO: Benchmarking Universal Single-Copy Orthologs; CNSA: China National Gene Bank Nucleotide Sequence Archive; Gb: gigabase; GO: Gene Ontology; kb: kilobase; KEGG: Kyoto Enyclopedia of Genes and Genomes; Mb: megabase; ML: Maximum Likelihood; NCBI: National Center for Biotechnology Information; stLFR: single tube long fragment reads; TE: transposable element.

## References

[1] Saitanu K Toxicity of the freshwater puffer fish *Tetraodon fangi* and *T. palembangensis* from Thailand. Toxicon, 1991; 29: 895–897. 1926188 10.1016/0041-0101(91)90226-h

[2] Subamia IW, Sudarto S, Purbowasito W, Sex determination in Indonesian pufferfish *Tetraodon palembangensis* Bleeker, 1852: implication for aquaculture and conservation. Indones. Aquac. J., 2011; 6: 37–45.

[3] Jaillon O Genome duplication in the teleost fish Tetraodon nigroviridis reveals the early vertebrate proto-karyotype. Nature, 2004; 431: 946–957. 15496914 10.1038/nature03025

[4] Sutaria D, Panicker D, Jog K, Sule M, Muralidharan R, Bopardikar I, Humpback dolphins (Genus Sousa) in India: an overview of status and conservation issues. Adv. Marine Biol., 2015; 72: 229–256. 10.1016/bs.amb.2015.08.006 26555628

[5] Hedges SB, The origin and evolution of model organisms. Nat. Rev. Genet., 2002; 3: 838–849. 12415314 10.1038/nrg929

[6] Fan G Initial data release and announcement of the 10,000 Fish Genomes Project (Fish10K). GigaScience, 2020; 9: giaa080. 32810278 10.1093/gigascience/giaa080 PMC7433795

[7] Zhang R Protocols for “Chromosome-level genome assembly of the humpback puffer, *Tetraodon palembangensis*”. protocols.io. 2021; 10.17504/protocols.io.bs8inhue. PMC9632004 36824331

[8] Chang L, Hi-C library preparation for the *Lateolabrax maculatus* genome. protocols.io. 2018l; 10.17504/protocols.io.ss4eegw.

[9] Julkowska M, Protocols for “RNA isolation with TRIzol”. protocols.io. 2018; 10.17504/protocols.io.pbndime.

[10] Wang O Efficient and unique cobarcoding of second-generation sequencing reads from long DNA molecules enabling cost-effective and accurate sequencing, haplotyping, and de novo assembly. Genome Res., 2019; 29: 798–808. 30940689 10.1101/gr.245126.118 PMC6499310

[11] Huang J BGISEQ-500 WGS library construction. protocols.io. 2018; 10.17504/protocols.io.ps5dng6.

[12] Huang J, BGISEQ-500 sequencing. protocols.io. 2018; 10.17504/protocols.io.pq7dmzn.

[13] Pederson ER, RNA and DNA extraction (Qiagen) of frozen tissue to nanopore sequencing. protocols.io. 2021; 10.17504/protocols.io.bj7tkrnn.

[14] Zhang R, Oxford Nanopore sequencing and library construction. protocols.io. 2021; 10.17504/protocols.io.btignkbw.

[15] Chen Y SOAPnuke: a MapReduce acceleration-supported software for integrated quality control and preprocessing of high-throughput sequencing data. Gigascience, 2018; 7: gix120. 29220494 10.1093/gigascience/gix120 PMC5788068

[16] Servant N HiC-Pro: an optimized and flexible pipeline for Hi-C data processing. Genome Biol., 2015; 16: 259. 26619908 10.1186/s13059-015-0831-x PMC4665391

[17] Li R The sequence and de novo assembly of the giant panda genome. Nature, 2010; 463: 311–317. 20010809 10.1038/nature08696 PMC3951497

[18] Vurture GW GenomeScope: fast reference-free genome profiling from short reads. Bioinformatics, 2017; 33: 2202–2204. 28369201 10.1093/bioinformatics/btx153 PMC5870704

[19] Luo R SOAPdenovo2: an empirically improved memory-efficient short-read de novo assembler. Gigascience, 2012; 1: 18. 23587118 10.1186/2047-217X-1-18 PMC3626529

[20] Xu M, Guo L, Gu S, Wang O, Zhang R, Peters BA, Fan G, Liu X, Xu X, Deng L, Zhang Y, TGS-GapCloser: A fast and accurate gap closer for large genomes with low coverage of error-prone long reads. Gigascience, 2020; 9(9): giaa094. doi:10.1093/gigascience/giaa094. 32893860 PMC7476103

[21] Walker BJ Pilon: an integrated tool for comprehensive microbial variant detection and genome assembly improvement. PloS One, 2014; 9: e112963. 25409509 10.1371/journal.pone.0112963 PMC4237348

[22] Liu X, Protocols for “the pipeline of Hi-C assembly”. protocols.io. 2018; 10.17504/protocols.io.qradv2e.

[23] Wataru K Integration of the genetic map and genome assembly of fugu facilitates insights into distinct features of genome evolution in teleosts and mammals. Genome Biol. Evol., 2011; 3: 424–442. 21551351 10.1093/gbe/evr041 PMC5654407

[24] Kang S Chromosomal-level assembly of *Takifugu obscurus* (Abe, 1949) genome using third-generation DNA sequencing and Hi-C analysis. Mol. Ecol. Resour., 2020; 20: 520–530. 31887246 10.1111/1755-0998.13132

[25] Zhou Y Chromosome genome assembly and annotation of the yellowbelly pufferfish with PacBio and Hi-C sequencing data. Sci. Data, 2019; 6: 267. 31704938 10.1038/s41597-019-0279-z PMC6841922

[26] Mandrioli M, Cuoghi B, Marini M, Manicardi GC, Cytogenetic analysis of the pufferfish *Tetraodon fluviatilis* (Osteichthyes). Chromosome Res., 2000; 8: 237. 10841051 10.1023/a:1009257131091

[27] Li F Morphological structure and karyotype of *Thamnaconus septentrionalis*. South China Fisheries Science, 2019; 15: 104–112.

[28] Benson G, Tandem repeats finder: a program to analyze DNA sequences. Nucleic Acids Res., 1999; 27: 573–580. 9862982 10.1093/nar/27.2.573 PMC148217

[29] Tarailo-Graovac M, Chen N, Using RepeatMasker to identify repetitive elements in genomic sequences. Curr. Protoc. Bioinformatics, 2009; 25: 4.10.1–4.10.14. 10.1002/0471250953.bi0410s25 19274634

[30] Meng G, Li Y, Yang C, Liu S, MitoZ: a toolkit for animal mitochondrial genome assembly, annotation and visualization. Nucleic Acids Res., 2019; 47: e63. 30864657 10.1093/nar/gkz173 PMC6582343

[31] Wataru I MitoFish and MitoAnnotator: a mitochondrial genome database of fish with an accurate and automatic annotation pipeline. Mol. Biol. Evol., 2013; 30: 2531–2540. 23955518 10.1093/molbev/mst141 PMC3808866

[32] Stanke M, Keller O, Gunduz I, Hayes A, Waack S, Morgenstern B, AUGUSTUS: ab initio prediction of alternative transcripts. Nucleic Acids Res., 2006; 34(suppl_2): W435–W439. 16845043 10.1093/nar/gkl200 PMC1538822

[33] Majoros WH, Pertea M, Salzberg SL, TigrScan and GlimmerHMM: two open source ab initio eukaryotic gene-finders. Bioinformatics, 2004; 20: 2878–2879. 15145805 10.1093/bioinformatics/bth315

[34] Burge C, Karlin S, Prediction of complete gene structures in human genomic DNA. J. Mol. Biol., 1997; 268: 78–94. 9149143 10.1006/jmbi.1997.0951

[35] Grabherr MG Full-length transcriptome assembly from RNA-Seq data without a reference genome. Nat. Biotechnol., 2011; 29: 644. 21572440 10.1038/nbt.1883 PMC3571712

[36] Pertea M, Kim D, Pertea GM, Leek JT, Salzberg SL, Transcript-level expression analysis of RNA-seq experiments with HISAT, StringTie and Ballgown. Nat. Protoc., 2016; 11: 1650. 27560171 10.1038/nprot.2016.095 PMC5032908

[37] Campbell MA, Haas BJ, Hamilton JP, Mount SM, Buell CR, Comprehensive analysis of alternative splicing in rice and comparative analyses with Arabidopsis. BMC Genomics, 2006; 7: 327. 17194304 10.1186/1471-2164-7-327 PMC1769492

[38] Doerks T, Copley RR, Schultz J, Ponting CP, Bork P, Systematic identification of novel protein domain families associated with nuclear functions. Genome Res., 2002; 12: 47–56. 11779830 10.1101/gr.203201 PMC155265

[39] Haas BJ Automated eukaryotic gene structure annotation using EVidenceModeler and the Program to Assemble Spliced Alignments. Genome Biol., 2008; 9: R7. 18190707 10.1186/gb-2008-9-1-r7 PMC2395244

[40] Elsik CG, Mackey AJ, Reese JT, Milshina NV, Roos DS, Weinstock GM, Creating a honey bee consensus gene set. Genome Biol., 2007; 8: R13. 17241472 10.1186/gb-2007-8-1-r13 PMC1839126

[41] Bairoch A, Apweiler R, The SWISS-PROT protein sequence database and its supplement TrEMBL in 2000. Nucleic Acids Res., 2000; 28: 45–48. 10592178 10.1093/nar/28.1.45 PMC102476

[42] Kanehisa M, Goto S, KEGG: kyoto encyclopedia of genes and genomes. Nucleic Acids Res., 2000; 28: 27–30. 10592173 10.1093/nar/28.1.27 PMC102409

[43] Harris MA, Clark J, Ireland A, Lomax J, Ashburner M, Foulger R, The Gene Ontology (GO) database and informatics resource. Nucleic Acids Res., 2004; 32(Suppl_1): D258–D261. 14681407 10.1093/nar/gkh036 PMC308770

[44] Jones P InterProScan 5: genome-scale protein function classification. Bioinformatics, 2014; 30: 1236–1240. 24451626 10.1093/bioinformatics/btu031 PMC3998142

[45] Li H TreeFam: a curated database of phylogenetic trees of animal gene families. Nucleic Acids Res., 2006; 34: D572. 16381935 10.1093/nar/gkj118 PMC1347480

[46] He Y A chromosome level genome of black rockfish, *Sebastes schlegelii*, provides insights into the evolution of live birth. Mol. Ecol. Resour., 2019; 19: 1309–1321. 31077549 10.1111/1755-0998.13034

[47] Edgar RC, MUSCLE: multiple sequence alignment with high accuracy and high throughput. Nucleic Acids Res., 2004; 19(32): 1792–1797. 10.1093/nar/gkh340 PMC390337 15034147

[48] Stamatakis A, RAxML-VI-HPC: Maximum likelihood-based phylogenetic analyses with thousands of taxa and mixed models. Bioinformatics, 2006; 22: 2688–2690. 16928733 10.1093/bioinformatics/btl446

[49] Yang Z, PAML: a program package for phylogenetic analysis by maximum likelihood. Bioinformatics, 1997; 13: 555–556. 10.1093/bioinformatics/13.5.555 9367129

[50] Sudhir K, Stecher G, Suleski M, Hedges SB, TimeTree: a resource for timelines, timetrees, and divergence times. Mol. Biol. Evol., 2017; 34: 1812. 28387841 10.1093/molbev/msx116

[51] Simão FA, Waterhouse RM, Ioannidis P, Kriventseva EV, Zdobnov EM, BUSCO: assessing genome assembly and annotation completeness with single-copy orthologs. Bioinformatics, 2015; 31: 3210–3212. 26059717 10.1093/bioinformatics/btv351

[52] Zhang R Genome data for the chromosome-level assembly of the humpback puffer, *Tetraodon palembangensis*. GigaScience Database. 2020; 10.5524/100755. PMC9632004 36824331

